# 

**Published:** 1918-10

**Authors:** 


### ANNOUNCEMENTS

### Sixty-Eight Vacancies in the Dental Corps, the United States Army

1. The Acting Surgeon General of the Army announces that there are, at the present time, sixty-eight vacancies in the Dental Corps, the United States Army, and that examinations for the appointment of dental surgeons will be held at various points in the United States, on Monday, November 4, 1918.

2. Application blanks and full information concerning these examinations can be procured by addressing "Surgeon General, U. S. Army, Washington, D. C."

3. The Dental Corps is a constituent part of the Medical Corps of the Army, and consists of officers in the grades of colonels, lieutenant-colonels, majors, captains and first lieutenants. Appointments therein are made at the rate of one for each 1,000 of the total strength of the Regular Army, authorized from time to time by law. Law requires that first lieutenants of the Dental Corps shall serve five years in that grade before being promoted, but for the period of the existing emergency this provision has been suspended by Act of Congress, and after one year's service as first lieutenant, a dental surgeon is eligible for promotion to the grade of captain, after which promotions are made in order of seniority as vacancies occur in the higher grades.

4. No applicant may under existing law be commissioned in the Dental Corps, unless he is between 21 and 32 years of age, a citizen of the United States, a graduate of a standard dental college, and of good moral character, nor unless he shall pass the usual physical examination required for appointment in the Medical Corps, and a professional examination which shall include tests of skill in practical dentistry and of proficiency in the usual subjects of a standard dental college course. Whether or not the applicant is married has no effect upon his eligibility for the Dental Corps.

5. Application for appointment must be made in writing to the Surgeon General of the Army, upon the prescribed blank form. All the interrogatories on the blank must be fully answered. In compliance with the instructions thereon, the application must be accompanied by testimonials, based upon personal acquaintance, from at least two reputable persons, as to the applicant's citizenship, character and habits.

The selection of the candidates is made by the Surgeon General from the applications submitted, and a formal invitation to report for examination to the most convenient examining board in each case will be issued by him.

6. The examinations are conducted under instructions from the Surgeon General and usually last six days. No allowances can be made for the expenses of applicants undergoing examination, whether incurred in travel to and from or during their stay at the place of examination, as public funds are not available for the payment of such expenses.

Each applicant, upon presenting himself to the board, will, prior to his physical examination, be required to submit his diploma as a graduate of a standard dental college. Should he fail to do so the examination will not proceed.

7. A first lieutenant receives $2,000 per annum; a captain, $2,400 per annum; a major, $3,000 per annum. These salaries are increased by ten per cent. for each period of five years until the maximum of forty per cent. is reached, excepting that the maximum salary of a major is $4,000 a year, and that of a lieutenant-colonel and colonel is $375 and $416.66 per month, respectively. In addition to their pay proper, they are furnished with a liberal allowance of quarters according to rank, either in kind, or where no suitable government building is available, by commutation. Fuel and light therefor are also provided. When traveling on duty an officer receives mileage for the distance traveled. On change of station he is entitled to transportation of professional books, and papers and a reasonable amount of baggage at government expense. Groceries and other articles for their own use may be purchased from the quartermaster at about wholesale cost prices. Dental surgeons are entitled to medical attendance and hospital treatment without charge other than for subsistence.

8. Officers of the Dental Corps are entitled to the privilege of retirement after forty years' service, or at any time for disability incurred in the line of duty. On attaining the age of sixty-four, they are placed on the retired list by operation of law. Retired officers receive three-fourths of the pay of their rank (salary and increase) at the time of retirement.

9. In order to perfect all necessary arrangements for the examination, applications must be in the possession of the Surgeon General at least two weeks before the date of examination. Early attention is therefore enjoined upon the intending applicants.

---

### Preparedness League of American Dentists

*Communication from the President.*—We are starting on our third year with the following officers elected at our annual meeting in Chicago on August 9, 1918:

President, J. W. Beach, Buffalo, N. Y.

Vice-President, J. D. Millikin, San Francisco, Cal.

Secretary, O. A. Oliver, Nashville, Tenn.

Treasurer, L. M. Waugh, New York. N. Y.

Representing the Surgeon General's Office, Lieutenant J. V. Gentilly, New York, N. Y.

Director General, W. D. Tracy, New York, N. Y.

We deeply regret the resignation of Dr. C. F. Ash as Director General, but activities in other directions made it imperative. He has been appointed a member of a committee for the conservation of platinum, an office which will occupy a large share of his time. His service as Director General has been of inestimable value to his country and to the League and a more efficient incumbent could not have been found. I know every member joins me in these sentiments, together with good wishes and the hope of long service to his government.

The assurance given by Dr. Ash that his interests are with the League at all times, also that his services will be given just as freely as before, has somewhat mitigated the effect of his resignation, but nevertheless, we shall greatly miss his active management of so important an office as that which he so ably occupied.

*The New Director General.*—Dr. Tracy, our new Director General, has been Director for the Department of the Northeast as well as Chairman of the New York Unit of the League. He is, therefore, especially qualified to assume the broader duties, inasmuch as he has been, as it were, the understudy of Dr. Ash.

No better selection could have been made following Dr. Ash and it gives me great pleasure and satisfaction to indorse him in every particular. This step means for him great sacrifice, but he has offered himself willingly and gladly to this great service. Every worker of the League will receive fullest co-operation from his department and I know he will receive equal return from our members and the benefits of our work will continue to increase.

We have a great work before us, for the League is developing into a real public service institution. We have at present no less than eight distinct objects, each of which demands special service from our members. The League is in position to direct all members of the profession to give full service to the great cause in our own special field. We have no need to look elsewhere for work to do.

Let each one of our army of 18,000 members become an ardent, conscientious worker in the vineyard of humanity, for the success of the colors and the triumph of justice and right.

*Colonel Logan and the League.*—In his President's address before a great audience of more than 5,000 people in the Auditorium Theater, Chicago, on the evening of August 6, last, Colonel Logan, as President of the National Dental Association, spoke in most appreciative terms of the service the members of the League have rendered in making the registrant dentally fit for military duties. We are deeply grateful for such expression, yet feel that our members are justly entitled to it. No one will ever know the amount of free service given by the dentists of America, not more than one-third of which will ever be recorded.

A suggestion was made by Colonel Logan that, inasmuch as the military camps are now well equipped, the Army dental department is in a position to do the necessary fillings for the recruit after entering the service; therefore, it is his desire that the League give special attention to reclaiming the dentally unfit in order to place them in Class A. By so doing, the dentist may render the greatest service to the Government by augmenting our fighting forces.

This suggestion is both timely and needed, particularly as the draft age has been extended to include a vast army of men who will need fixed bridgework to put them in the firing line. To do this means time, money and sacrifice for all of us, but we *will do it* without question or complaint. It is our duty and no right-thinking dentist can side-step the issue.

Continue doing all necessary fillings and extractions as heretofore, for there is more dental work needed by our soldiers than could be done if *every one* of the 50,000 dentists of the United States should devote his whole time to this purpose. Shoulder the additional burden manfully and cheerfully for the sake of our own boys who are laying down their lives by the thousands that we may spend our declining years in our own peaceful country and under the protection of the flag that shall bring enduring democracy to the whole world.

Assure Colonel Logan of our co-operation in this way.

*The Wheels of the League.*—The Dental Motor Car exhibit in Grant Park, opposite the Auditorium Hotel, during the meeting of the National Dental Association at Chicago, proved to be one of the most interesting features of the convention. Chairman Weaver secured a host of friends for the car and its future usefulness is assured. The Red Cross soon will begin shipping them overseas for active service at the front.

It remained, however, for Dr. Ottolengui to again demonstrate his resourcefulness and originality of thought by inaugurating a plan for securing a Dodge motor car for the special use of the dental officers in each military camp. This movement is much needed, as a motor car will greatly increase the usefulness of the officers by transporting them rapidly on their daily round of duties.

Through his activity, ably assisted by Dr. F. M. Casto, more than $1,100 was raised in less than one hour at the meeting of the American Society of Orthodontists and he immediately ordered a car delivered at Camp Greenleaf. Another one has been ordered for Camp Upton and more will follow as rapidly as possible. These cars will be presented through the League and will pass from commandant to commandant as changes occur in the camps.

*Our Study Course.*—One of the most urgent duties of the League is to make available a course in war oral and dental surgery for the civilian practitioner. We are exerting every effort to have it ready as soon as possible. Some delay has been caused by unavoidable circumstances, but League members may be sure no further delay will occur.—J. W. Beach, President.

---

*Communication from Director General Tracy.*—While it is true that as the Dental Corps of the United States Army increases in numbers and efficiency the need for volunteer services on the part of the Preparedness League in connection with filling operations will correspondingly be reduced, it is not intended that the activities of the League shall be curtailed in this respect at present, as the accession of men from the new draft will be greater, both as to numbers and rapidity of induction than heretofore. It is, on the contrary, absolutely necessary that greater impetus and effort be given to our work.

*Bridgework.*—It is also desirable that the scope of our work should be broadened to include the restoration of those registrants who are physically fit for general military service, and are held in Group C solely because of their dental deficiency.

A large percentage of these cases can be restored by the insertion of small bridges, thus bringing them up to the minimum dental requirements of six masticating teeth in occlusion, and six incisive teeth in occlusion. No extensive restorations by bridgework are contemplated. In other words, only those cases are recommended for treatment which can be brought up to minimum requirements by the insertion of a small and inexpensive bridge.

At present there is no government regulation compelling registrants in the class mentioned to have this work done at their own expense, and no provision exists making it possible for them to have this work done at the cantonments.

Most of the men mentioned above are anxious to be made dentally fit in order that they may be inducted into general military service and thus be of use to their country in the fighting line. I have found that with few exceptions the local boards, throughout the country, are very appreciative of the dental services which have been rendered to the registrants under their control, by the members of the Preparedness League of American Dentists, and I am sure if it is known that we have a large list of volunteer dentists who have offered to take cases of the type outlined above, a great number of registrants now standing in Group C can be restored and immediately transformed to Class 1-A.

It is, therefore, plainly the duty of every member to write to the League officer in charge of the work in his section notifying him that he will be glad to take one, two or more cases each month, without expense to the registrant, the League or the government.

*Families of Soldiers.*—Our activities should also embrace the care of dependent families of our soldiers, sailors and marines who are unable to pay for dental services.

It has also been stated that one of the greatest possibilities for service on the part of the Preparedness League and its members would be a full and free cooperation between the League and the Home Service Section of the Red Cross.

In many instances the wives and families of soldiers in the United States Army who have been accustomed to private dental treatment, but who, because of the reduction in their incomes can not now afford to go to a private practitioner, should be taken care of by the Volunteer Dentists of the Preparedness League.

The Home Service Section of the Red Cross, through their authorized agents, will investigate each case as presented and in those cases recommended for dental treatment at the hands of the Preparedness League, the patient will receive a card bearing the endorsement of the Red Cross, stating that the patient is a member of a soldier's family and worthy of free dental treatment.

In this manner much suffering can be alleviated and much dental trouble prevented among the families of the men who have gone forward to defend our country.

The medical profession is already co-operating most generously with the Home Service Section of the Red Cross and it is hoped that every member of the P. L. A. D. will share in this work.

This type of work has already been begun by a number of Preparedness League Units in various states and the pleasure and satisfaction which the members are finding in taking care of these cases warrant us in believing that it can be extended to every state in the Union.

Plans are now being formulated to take care of this new department in our activities, and it is the intention of the officers of the League to have the work so arranged as to bring no special hardship on any one member.

While the Allied forces are meeting with gratifying success at the front and ultimate victory is assured, this is no time for relaxation in any of our war activities or patriotic efforts and it is only by putting every ounce of human energy into action that the result desired by all truly civilized peoples can be attained.

As a national patriotic body of professional men we are strong and well organized, but in a few states the work has developed slowly and it is urged that in such states the directors and officers grasp anew the great possibilities of the League's work and take up with increased determination the duties they have assumed.

Assuring you of my desire to assist and co-operate with you in every possible manner, I am, Yours very truly, W. D. Tracy, Director General for the United States.

---

*Communication from the Committee on Motor Cars for Camps.*—The undersigned visited Camp Greenleaf this summer. Camp Greenleaf adjoins Fort Oglethorpe, Ga., and is some six or eight miles from Chattanooga, Tenn. Here are situated training schools for medical men, dentists and others. It is with the Dental Training School, of course, that we are most interested. The writer quickly discovered that the dental corps at each military camp is sadly in need of means of rapid transportation about the camp, and to the neighboring city. Military camps accommodate from 25,000 to 75,000 men. Cities of that size would have trolley cars, cabs and other means of transportation. Moreover, there would be numerous shops. At a military camp there is but one place for procuring any needed article, and all such places are widely scattered. It is manifest that an automobile would greatly add to the efficiency of the dental corps at each camp, by saving time that would otherwise be expended in walking great distances. This is so true that many officers have purchased second-hand Ford or Dodge cars, but these being private property must be maintained at the officers' individual expense. Bills for gasoline, tires, repairs, etc., make large holes in an officer's pay check. This seems hardly fair.

The writer, therefore, conceived the idea that the Preparedness League should procure a car for Camp Greenleaf. On his return to New York he reported this to Director General Ash, and was surprised to learn that a visit to Camp Upton had impressed Dr. Ash with the same need, and he was just inaugurating a campaign for obtaining funds for that purpose. The writer was then appointed chairman of a committee to foster this movement. Drs. Ash and Tracy sent out a circular appeal to the dental profession in New York, and through the generous response that resulted a Dodge car has been presented to the Dental officers at Camp Upton.

The writer sent out a circular letter to one hundred men throughout the country asking for contributions to a fund to purchase a car for Camp Greenleaf. Before replies could have been expected to come in, the meeting of the American Society of Orthodontists convened in Chicago, and Dr. F. M. Casto, Secretary of that Society, and an officer in the League, suggested that an appeal be made in open meeting for contributions. This was done, with a response that redounds to the credit, loyalty and generosity of the members of that organization, not overlooking a few guests who were present. $1,131 was subscribed and paid in, in just thirty-eight minutes. A Dodge Touring Car was purchased by telegraph from a firm in Chattanooga, and was promptly delivered and is in use at the camp. A quotation from a letter of thanks received from one of the dental officers places an aspect upon this work which is important. It is as follows:

"When the high officers of the camp see our car, and when the instructors in the Sanitary, Cardio-vascular, T. B., X-Ray, Orthopedic and other schools see it they know that the members of our profession are backing us for all they are worth and that we mean business. All such things serve to drive dentistry higher and higher in the estimation of the men around us and through these things, to stimulate us, we hope, to drive so hard that some day when the old standards of dentistry emerge from the war clouds, their colors will be flying high and Uncle Sam and the whole world will turn to our profession and say: "Well done, good and faithful servants."

*Future Efforts.*—The League may not be able to supply cars for all the military camps, but we are undertaking to furnish cars for at least ten more. We are at the moment of preparing this for publication awaiting the decision of the authorities as to which camps are most in need, whereupon the campaign will be pushed. It may be announced, however, that as soon as these camps are selected, the state directors will be advised and asked to co-operate in collecting funds towards the car for the nearest camp.

After paying for the two cars already purchased and delivered, we are pleased to report that we already have a goodly sum left in the treasury of this fund. Very truly, R. Ottolengui, Chairman, Committee on Motor Cars for Camps.

